# Using Odontoblasts Derived from Dog Endometrial Stem Cells Encapsulated in Fibrin Gel Associated with BMP-2 in a Rat Pulp-Capping Model

**DOI:** 10.3390/cimb45040196

**Published:** 2023-04-03

**Authors:** Elham Hoveizi, Hadi Naddaf, Sina Ahmadianfar, Sara Bernardi

**Affiliations:** 1 Department of Biology, Faculty of Science, Shahid Chamran University of Ahvaz, Ahvaz 6135783151, Iran; 2 Department of Clinical Sciences, Faculty of Veterinary, Shahid Chamran University of Ahvaz, Ahvaz 6135783151, Iran; hdnaddaf@scu.ac.ir (H.N.); s-ahmadianfar@phdstu.scu.ac.ir (S.A.); 3 Department of Life Health and Environmental Sciences, University of L’Aquila, 67100 L’Aquila, Italy

**Keywords:** bone morphologic protein-2 (BMP-2), canine endometrial stem cells, dental tissue engineering, pulp-capping

## Abstract

This study aimed to treat dental injuries by utilizing one of the most advanced tissue engineering techniques. In this study, an in vitro model was employed to investigate the proliferation and odontogenic differentiation of canine endometrial stem cells (C-EnSCs). Furthermore, the dentin regeneration potential of odontoblast like-cells (OD) derived from C-EnSCs was assessed in rats. The C-EnSCs were isolated by the enzymatic method and identified by flow cytometry. The C-EnSCs were encapsulated in fibrin gel associated with signaling factors to create the proper conditions for cell growth and differentiation. Then, the OD cells were associated with bone morphologic protein-2 (BMP-2) to promote dentin formation in vivo. The animal model used to evaluate the regenerative effect of cells and biomaterials included the preparation of the left maxillary first molar of rats for direct pulp capping operation. Animals were divided into four groups: group 1, a control group without any treatment, group 2, which received fibrin, group 3, which received fibrin with ODs (fibrin/ODs), and group 4, which received fibrin with ODs and BMP-2 (fibrin/ODs/BMP-2). The morphological observations showed the differentiation of C-EnSCs into adipose, bone, neural cells, and ODs. Furthermore, the histomorphometric data of the treated teeth showed how fibrin gel and BMP2 at a concentration of 100 ng/mL provided an optimal microenvironment for regenerating dentin tissue in rats, which was increased significantly with the presence of OD cells within eight weeks. Our study showed that using OD cells derived from C-EnSCs encapsulated in fibrin gel associated with BMP2 can potentially be an appropriate candidate for direct pulp-capping and dentin regeneration.

## 1. Introduction

Teeth disorders can cause serious problems in muscle function, nutrition, speech, and facial beauty, and even cause problems in personal and social life [1]. Preventive measures in dentistry are fundamental since a delay of treatment can lead to the occurrence of halitosis, dysphagia, hypersalivation, ptyalism, bleeding gums, eventual tooth loss, edentulism, occlusion instability, and low quality of life [2,3].

Dental hard tissues are known to be extremely important since their regeneration does not occur. Enamel is a tissue without cells, and dentin regeneration often appears disorganized.

Tissue engineering has been explored to serve as a viable alternative for treating tooth lesions and loss of dental tissue; in particular, in regenerating the natural structure of teeth. It is fundamental that an extracellular matrix-mimicking scaffold, progenitor/stem cells, and morphogens are integrated to realize the potential of regenerative treatments for teeth [4]. Tissue engineering techniques applied to regenerative treatment for dental tissue include stem cell cultures that can be differentiated into target tissues on a three-dimensional medium to provide a suitable growth network, a scaffold, which plays a crucial role in tissue engineering [5,6]. In addition, it is possible to exploit endogenous and non-endogenous molecules to guide the differentiation through epigenetic modifications of some lines of stem cells for targeted regenerative purposes [7,8]. In the development of suitable scaffolds, the use of natural materials has been increasing for years. Fibrin is a fibrous biopolymer that is formed by the polymerization of fibrinogen during blood clotting. From this point of view, fibrin-based scaffolds are considered one of the most suitable options because they possess the characteristic of a standard bioactive scaffold; they are also autologous and can be obtained from the patient, providing clinical advantages, such as easy obtainment, high biocompatibility, and minimally invasive surgery [9,10].

In the last few years, endometrial stem cells (EnSCs) have been identified as a source of mesenchymal stem cells (MSCs) [11]. In addition, stem cells isolated from canine endometrium express the characteristics of MSCs and self-renewal capability. The ease of access, differentiation capabilities, lack of ethical concerns, and abundant availability of canine En-MSCs make them an attractive alternative source of adult stem cells for future research and clinical application in veterinary science [12].

With the advancement of tissue engineering, the use of a variety of appropriate signaling factors, along with scaffolding and cells as a third pillar, can be very valuable. Among these growth factors, bone morphologic protein-2 (BMP-2), which belongs to the transforming growth factor β (TGF- β) family, plays an essential role in the growth and regeneration of skeletal tissues and has shown odontogenic and osteogenic properties, both in vitro and in vivo [13,14]. In addition, BMP-2 stimulates the differentiation of dental pulp stem cells (DPSCs) into odontoblasts [15].

The aim of this study was to investigate, in an in vitro model, the proliferation and odontogenic differentiation of canine endometrial stem cells (C-EnSCs) and their use, encapsulated in fibrin gel and BMP- 2, in the regeneration of hard dental tissue.

## 2. Materials and Methods

All the study procedures were performed according to the Animal Care and Use Committee (ACUC) of the Shahid Chamran University of Ahvaz with the code EE/99.3.02.47192/scu.ac.ir.

### 2.1. Cell Isolation, Culture, and Identification

After an ovariohysterectomy on a healthy 1-year-old dog, a biopsy from the uterine endometrium was performed to isolate C-EnSCs. The sample was placed in a tube containing Hank’s medium (Gibco, Waltham, MA, USA) containing 5% penicillin-streptomycin (Gibco, USA). The tissue sample was cut into small pieces after several washing cycles. The crushed pieces were added to a falcon plastic tube containing type 1 collagen enzyme (Sigma, Ronkonkoma, NY, USA) at a 2 mg/mL concentration and incubated for two hours at 37 °C in a CO_2_ incubator (Sina, Hombarat, Iran). In the next step, DMEM/F12 (Dulbecco’s Modified Eagle Medium/Nutrient Mixture F-12; Gibco, USA) with 10% fetal bovine serum (FBS; Gibco, USA) was added to each sample and pipetted. To remove undigested tissues, the resulting solution was passed through a 70 μm mesh once and a 40 μm mesh twice. Blood cells were also removed with Ficoll (Sigma, USA). Isolated cells were transferred into DMEM/F12 medium supplemented with 15% FBS and streptomycin-penicillin 1%. Then, three passages were performed with Trypsin-EDTA (Gibco, USA) [16].

### 2.2. Differentiation of C-EnSCs into Adipose, Bone, and Nerve Cells

The collected cells were developed into adipocytes, osteocytes, and nerve cells to demonstrate their pluripotency. Approximately 1 × 10^4^ cells per well of third passage cells were treated for three weeks with the adipogenic and osteogenic medium in 24-well plates. For adipogenic media, DMEM/F12 + ascorbic acid (50 g/mL), dexamethasone (10^−7^ M), indomethacin (50 g/mL), and 10% FBS were used. As the osteogenic media, DMEM/F12 medium containing ascorbic 3-phosphate (50 g/mL), dexamethasone (10^−8^ M), beta-glycerol phosphate (10 mM), and 10% FBS were used. Alizarin red staining was used to detect differentiation into osteocytes, while oil red O staining was used to detect differentiation into adipocytes. To differentiate C-EnSCs into neurons, the cells were cultured in 24-well plates at 5 × 10^4^ cells/well. The cells were cultured in DMEM/F12 medium with bFGF (20 ng/mL), EGF (10 ng/mL), and 5% FBS for 14 days. As a control sample (undifferentiated cells), the culture dish was kept in DMEM/F12 medium with 10% FBS. After 14 days, immunocytochemical tests were conducted using neuron-specific antigens (Nestin). Additionally, the nuclei of the cells were stained with 1 µg/mL DAPI fluorescent dye [17].

### 2.3. Flow Cytometric Analysis

Following centrifugation at 2000 RPM for five minutes, the cells were washed twice with sterile PBS, 100 microliters of which were added into 1.5 mL Eppendorf tubes (106 cells). With appropriate control, the conjugated antibodies to CD90, CD105, CD34, and CD45 were added in a dark environment. The sample was chilled for an hour before being added to 100 microliters of 1% cold paraformaldehyde and kept in the refrigerator without exposure to light until flow cytometry was performed.

### 2.4. Differentiation of C-EnSCs into Odontoblasts

The cells from the third passage were cultured for 21 days in differentiating medium containing DMEM/F12, FBS (10%), beta glycerol phosphate (5 mM), dexamethasone (0.1 μM), and ascorbic acid (50 μg/mL; Sigma). To assess the differentiation of C-EnSCs into odontoblasts and the expression of dentin sialophosphoprotein (DSPP) and dentin matrix protein-1 (DMP1) genes by qRT-PCR, alizarin red staining (ABI, Los Angeles, CA, USA) was used [18].

### 2.5. cDNA Synthesis and RT-PCR Analysis

A real-time PCR test was used to detect the expression of odontoblastic markers, including DMP1 and DSPP, at the mRNA level. Hence, 2.5 × 10^5^ cells were seeded and differentiated in each well of a 6-well plate (SPL, Pocheon-si, Korea). RNA was extracted using a kit (Cinnagen, Tehran, Iran). The concentration and purity of the extracted RNA were measured with a nanodrop device (Thermo, Waltham, MA, USA). cDNA was synthesized using the SuperScript First-Strand Synthesis kit (SinaClon, Tehran, Iran). A real-time PCR test was performed using the Amplicon kit and RUSH device (Rush, Heidelberg, Germany), and the Actinβ gene was used as an internal control. Primer sequences were also designed using the NCBI reference site (Table 1).

### 2.6. Preparation of Hydrogel-Cell Complex

For the preparation of the fibrin hydrogel scaffold, M199 medium (Gibco, USA) 1% penicillin-streptomycin (Gibco, USA) and fibrinogen powder (Sigma, USA) with a concentration of 3 mg/mL were used. First, the cell scaffold (M199 medium containing FBS and pen-strep + fibrinogen at a concentration of 3 mg/mL) and BMP-2 with a concentration of 100 ng/mL were poured into each well and then 5 × 10^4^ cell suspension was added and aspirated gently to prevent bubble formation. Then, 15 µL of thrombin at a concentration of 120 U/mL were added to 500 µL of hydrogel in each well and were immediately gently aspirated. The culture medium was gelatinized, and the cell culture plate was transferred to the incubator. After 2 h of incubation of the cells on the hydrogel scaffolds of each well, 1 µL of M199 medium containing 10% FBS serum and 5% antibiotic was added and transferred to the incubator again [10].

### 2.7. Scanning Electron Microscopy Analysis

The cells were cultured in fibrin hydrogel scaffolds for 5 × 10^4^ cells for 2 days for scanning electron microscopy. Then, to the pits containing 300 µL of the gel, 2.5% glutaraldehyde solution was added and kept at 25 °C for 2 h, then washed twice with 300 µL of PBS. With increasing concentrations of alcohol (30, 50, 70, 80, 90, and 100%), dehydration steps were performed. The scaffolds were then freeze-dried (Crist-Germany), covered with gold particles, and photographed by scanning electron microscopy (Philips-Netherlands) at different magnifications and at 20 Kv.

### 2.8. Evaluation of Cell Survival by Acridine Orange/Ethidium Bromide (AO/EB) Staining

The cells (5 × 10^4^ cells/well) were cultured in fibrin gel for 24 and 72 h and stained with acridine orange/ethidium bromide at a concentration of 100 µg/mL (1:1) for 10 min and monitored using a fluorescence microscope (Olympus, Tokyo, Japan). Here, the live and dead cells were observed as green and orange, respectively.

### 2.9. BMP-2 Loading and Release Behavior in Fibrin Gel

As mentioned, BMP-2 was added at a concentration of 100 ng/mL in the hydrogel. The purified hydrogel was utilized as a control. The samples were kept at 37 °C for the following investigations. To investigate the release of BMP-2 from the fibrin gel, PBS was picked as a buffer solution. In total, 1 mL of PBS was added to the 24-well plate and kept at 37 °C in an incubator for 28 days. At time points 1, 3, 7, 14, 21, and 28 days, the supernatant was collected, and the concentration of BMP-2 was estimated by ELISA [15].

### 2.10. MTT (2,5-Diphenyl-2H-tetrazolium Bromide) Assay

To determine the effects of BMP-2 with concentrations of 50, 100, 150, and 200 ng/mL on the viability of C-EnSCs cells, the MTT test (Sigma, USA) was used. The MTT test was performed on C-EnSCs cells treated in 96-well plates within 24 h. In this assay, 10 μL of MTT solution and 90 μL of DMEM/F12 medium (Gibco, USA) without FBS (9:1) were added to each well. Cells were incubated for four hours. In the next step, 100 μL of DMSO (Merck, Darmstadt, Germany) were added to the well as a solvent for the formazan crystals, and the absorbance was measured at 570 nm using an ELISA reader (Fax 2100, USA). The percentage of cell viability was calculated based on the following formula:

Cell viability percentage (Viability%) = (treatment group OD/control group OD) × 100.

### 2.11. Preparation of the Teeth and Design of Models

Twenty male Wistar rats weighing 230 ± 30 g, aged 200 to 250 days, were obtained from the animal care centers laboratory of Ahvaz Jondishapur University of Medical Sciences. Rats were kept in solitary confinement during this experiment under the same conditions. The animals were anesthetized with ketamine (10%, 100 mg/kg) and xylazine (2%, 10 mg/kg) administered intraperitoneally. A pulp capping procedure was performed on the maxillary first molar. In the following step, the enamel and dentin were contoured with special glasses with a magnification of 4.5, a micromotor handpiece, and a cylindrical diamond bur (HAGER, Blieskastel, Germany). After the enamel and dentin were removed, the pulp chamber roof was perforated using a catheter tip [19].

### 2.12. Transplantation of Stem Cells and Grouping

Each group of five received appropriate treatment after preparing the cavity. As a negative control group, group 1 received no treatment, and the cavity was only covered with a glass ionomer cement. In group 2, fibrin and glass ionomer operations were performed. The tooth in group 3 was filled with fibrin, OD cells, and glass ionomer; while in group 4, fibrin was used with OD cells, BMP-2, and a glass ionomer sealant. The antibiotic enrofloxacin was provided as 0.5 mL in 500 mL of drinking water daily for three days to prevent infection. Morphine analgesia was administered intramuscularly at a 3 mg/kg dose to alleviate the animal’s postoperative pain. A soft diet was implemented to prevent injury to the operating site.

### 2.13. Analyzing Histomorphometric Data

Following euthanasia after eight weeks, the samples were fixed for one week in 10% formalin buffer. In order to decalcify the teeth, they were immersed for 14 days in an ethylenediamine tetraacetic acid (EDTA) solution (Merck, Germany). Lastly, hematoxylin and eosin (H&E) stains were carried out after embedding and preparing tissue sections (5 µm). Light microscopy (Olympus, Japan) was used for imaging. Dentin bridge formation, bridge thickness, continuity and location of the dentin bridge, presence and distribution of blood vessels at the site, pulp status, and presence and position of the odontoblast layer (using Image J software, 1.8.0_172) were all observed and compared.

### 2.14. Cell Tracker Staining

In the staining process with DiI dye, C-EnSCs were exposed to DiI dye at concentrations of 0.5 μg/mL before transplantation and incubated for 40 min. Then, the supernatant was withdrawn, and the cells were washed two times with PBS.

### 2.15. Statistical Analysis

SPSS software (Norman, Cleveland, OH, USA, code: 546467) was used to perform statistical analysis to assess any difference in the genetic expression of the odontoblastic markers and the viability of the cells exposed to the experimental scaffold. The used tests were one-way ANOVA with post hoc Tukey. The software Excel 2019 was used to create the graph. A significant difference between the samples was defined as *p* ≤ 0.05.

## 3. Results

### 3.1. Isolation, Morphology, and Identification of C-EnSCs

Isolated cells from canine uterine endometrium appeared morphologically elongated and spindle-shaped, possessed a fibroblast-like shape, and were able to adhere strongly to the flask bottom. A few non-specific cells were present in the early phases of cell extraction, which were removed in the subsequent passages, leaving only specific cells with a spindle-shaped morphology in the third passage. Additionally, cells had a strong tendency to proliferate and bind to neighboring cells.

**Figure 1:**
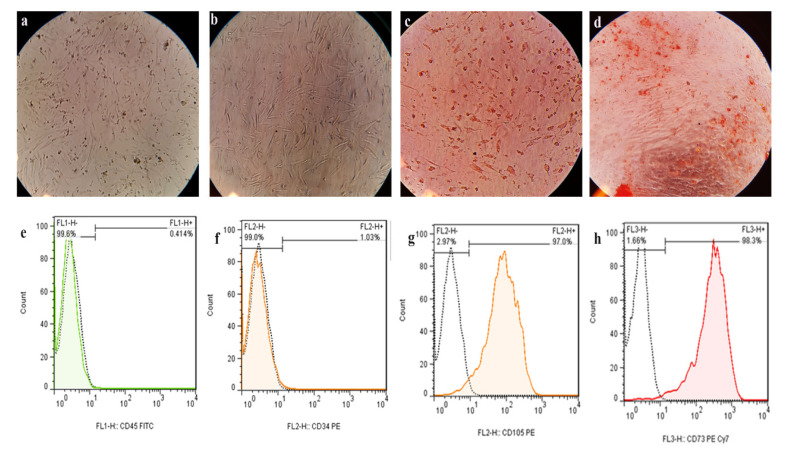
Inverted microscopy was used to examine the morphology of C-EnSCs. (**a**) C-EnSCs after the first culture. (**b**) C-EnSCs after the third culture. (**c**) C-EnSCs that differentiated into osteoblast cells and calcium storage deposits are marked in red. (**d**) C-EnSCs that differentiated into adipocytes after 21 days of treatment. Adipose vesicles are red. (**e**–**h**) Evaluation of C-EnSCs using flow cytometry after the third passage. (**e**) The expression level of the CD34 marker was about 0.41%. (**f**) The marker CD45 was expressed at about 1.03%. (**g**) The marker CD105 was expressed at about 97%. (**h**) CD90 marker was expressed at about 98.3%.

The differentiation capabilities of the C-EnSCs were evaluated in vitro after three passages. Adipogenic differentiation of C-EnSCs to adipocytes was confirmed by oil red O staining and inverted microscopy on day 21 of adipogenic differentiation. In this study, a specific nestin neural stem cell marker was examined. The nestin neuro markers expression was validated by fluorescent labeling, which supported the neurological nature and purity of the cells as compared to the control group. In the osteogenic media, C-EnSCs developed into osteoblasts within 21 days. Alizarin red staining and inverted microscope observations of red calcium deposits indicated osteoblast development (Figure 1a–d).

### 3.2. Flow Cytometry Results

Surface protein markers are one of the most important methods for determining the phenotype of mesenchymal stem cells after isolation. Cells derived from uterine endometrium (in the third passage) express the surface markers CD90 and CD105. The cells expressed the markers of CD90 and CD105 at 97% and 98.3%, respectively. The markers of CD34 and CD45 were expressed at 0.414% and 1.03% (Figure 1e–h).

### 3.3. Odontogenic Differentiation Analysis Using Alizarin Red Staining and qRT-PCR

Inverted light microscopy observation confirmed differentiation into OD cells by alizarin red staining and red calcium deposits at day 21 of differentiation.

**Figure 2:**
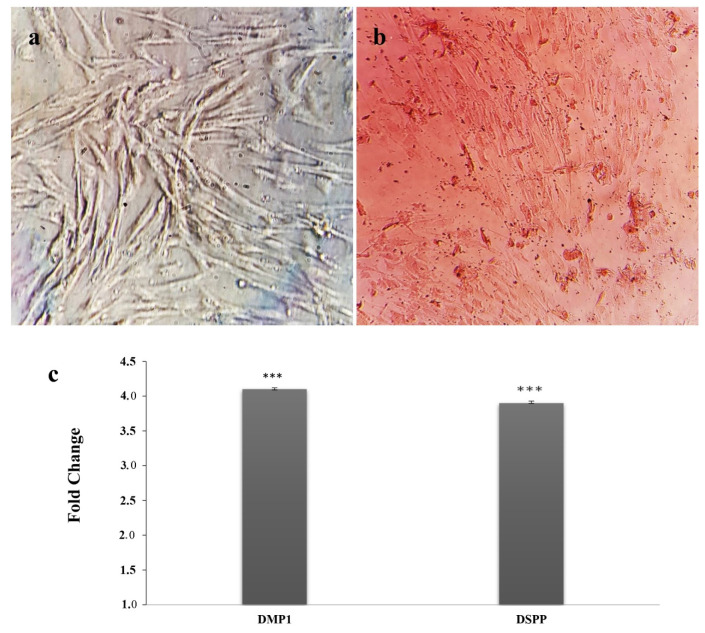
(**a**) The control sample. (**b**) Examining the differentiation of C-EnSCs into odontoblast, 21 days after differentiation. C-EnSCs that have differentiated into odontoblast cells and calcium storage deposits are marked in red. (**c**) Evaluation of expression analysis of DMP1 and DSPP genes after 21 differentiations in an odontogenic environment at mRNA level using qRT-PCR. The experiment was repeated at least three times, and the normalizing gene was Actin-B. *** shows a significant difference with the control group at the level of *p* < 0.001.

Based on the qRT-PCR results, the expression level for the DMP1 gene was 4.6-fold higher than the control group (Group 1; *p* ≤ 001). DSPP gene expression was 4.3-fold higher than the control sample (*p* ≤ 001) (Figure 2).

### 3.4. SEM Observations of Fibrin Gel

**Figure 3:**
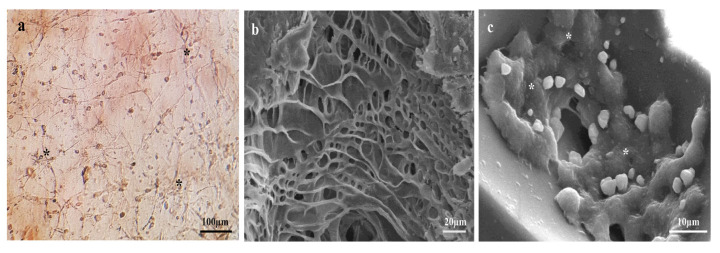
(**a**) An inverted microscopy picture of C-EnSCs cultured in fibrin gel. (**b**) SEM photographs as a 3D open porous and interconnected porosity. (**c**) C-EnSCs attached and spread in the fibrin hydrogel. Stars indicate the location of cells in the gel.

The SEM images of the fibrin allowed us to assess how the scaffold produced a uniform, regular porous, and fibrous gel with interconnectivity (Figure 3). In Figure 3b, the SEM microphotographs show the C-EnSCs in the fibrin gel after drying. In addition, the SEM photographs indicate suitable cell attachment and perfect integrity between the cells and the fibrin gel (Figure 3).

### 3.5. Viability Assessment

**Figure 4:**
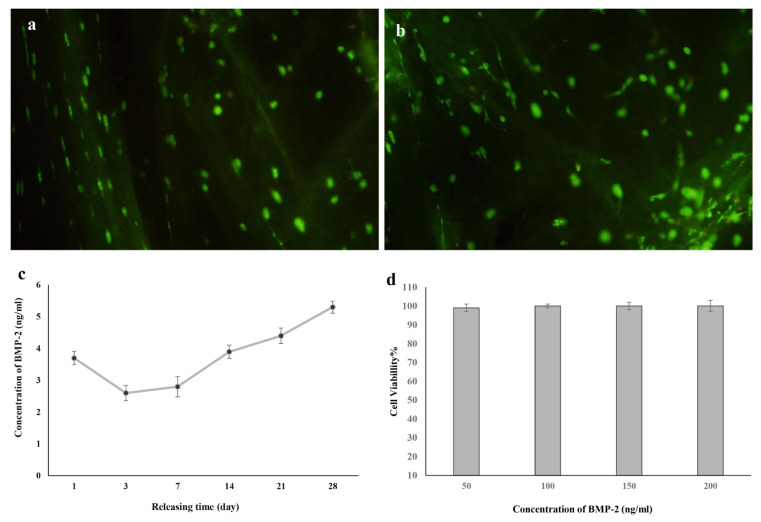
Fluorescence microscopic images of C-EnSCs in fibrin gel showing green staining for viable cells with 100 ng/mL concentration of BMP-2 (10X). (**a**) After 1 day (**b**). After 3 days. (**c**) Release of BMP-2 from fibrin gel. Amount of BMP-2 release per time point is graphed. (**d**) Results of the viability percentage of C-EnSCs using MTT 24 h after BMP-2 treatment. At concentrations of 50, 100, 150, and 200 ng/mL, the viability of C-EnSCs was 99–100% in all concentrations.

The results of AO/EB staining on the first and third days showed the survival of cells cultured in fibrin gel containing the BMP-2, and as shown in Figure 4a,b, almost all cells appeared shining green. Additionally, according to the results of the MTT assay, at concentrations of 50, 100, 150, and 200 ng/mL BMP-2, the viability of C-EnSCs was 100%, and these concentrations of BMP-2 had no toxic effect on cells. Therefore, there was no statistically significant difference between the concentrations used for BMP-2. In this experiment, a concentration of 100 ng/mL BMP-2 was used (Figure 4a–c).

### 3.6. Release Behavior of BMP-2

The release behavior of BMP-2 from hydrogel was stimulated using the ELISA investigation. The quantity of BMP-2 concentration was calculated using a linear curve. For BMP-2 releases, an initial rapid release achieved equilibrium at 1 day and then persisted in a stable method for up to 28 days. According to the consequences, 3.7 ng/mL of BMP-2 were released from the hydrogel on 1 day, and the accumulative release achieved 5.3 ng/mL at 28 days (Figure 4d).

### 3.7. Results of Histomorphology and Histomorphometry Using H&E Staining

**Figure 5:**
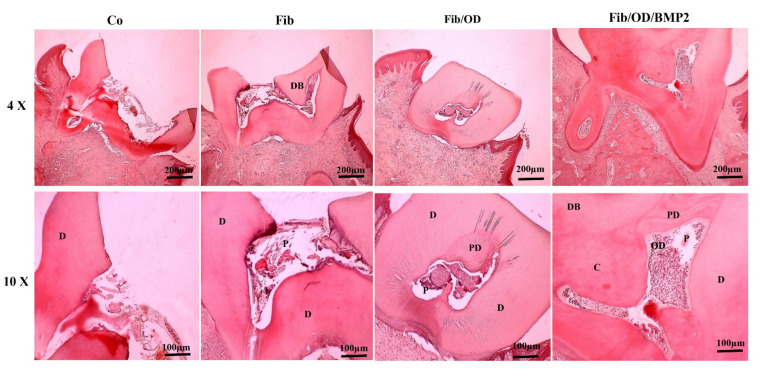
Analysis of the histomorphological results of the first molar by hematoxylin-eosin staining after 8 weeks for various groups by magnification order. DB: dentin bridge, D: dentin, P: pulp, PD: predentin, OD: odontoblast, V: blood vessel, C: calcification.

After eight weeks, histomorphology and histomorphometry studies were performed in different groups. We examined the dentin bridge, odontoblast cells, loose connective tissues, dentin and predentin, calcification, and blood vessel formation. Dentin is created from very thin layers (fragile layers of predentin). In this study, no dentin bridge appeared in the control group samples. ODs were not found in a single layer or as a single cell. The control group showed no signs of loose connective tissue. The samples in this group had no calcification or blood vessel development. The dentin bridge appeared partially developed on the fibrin group specimens while the margins were untouched, but at a higher magnification, the dentin bridge appeared broken (Figure 5). This group did not have an odontoblast layer or odontoblast cells. However, there was loose tissue in the pulp. At higher magnification, most dentin tubules were found, but there were no lacunae. Blood vessels in this group were atrophic, but no calcification was detected. The dentin bridge in the fibrin/ODs group samples was dense and continuous. On the other hand, the odontoblast cells were not observed as a layer but rather as a smattering. There was still loose connective tissue in the pulp. More searching led to the discovery of dentin formation, tubules, and lacunae. Calcification and blood vessel formation also occurred. The samples from the group of fibrin/PDs/BMP-2 showed complete dentin bridges with high thickness and continuity. Unlike other groups, this did not contain layered and enclosing odontoblast cells. There was no loose connective tissue or only a small amount. Dentin development, tubular layers, and lacunae were all observed. Calcification occurred, and there were many blood vessels in the restored area (Figure 5).

### 3.8. The Thickness of the Dentin Bridge

According to the dentin bridge area findings and blood vessel creation across groups, the fibrin/ODs/BMP-2 group ranked the highest, followed by the fibrin/ODs, fibrin, and finally, the control group.

Dentin bridge thickness was also categorized into three groups: thick (more than 250 microns), medium (150–249 microns), and thin (less than 150 microns). Fibrin/ODs/BMP-2 was therefore classified as thick. In the fibrin/ODs group, the thickness was classified as medium. The fibrin group had a thickness of fewer than 149 microns, making it thin. There was no dentin bridge in the control group (Table 2).

The fibrin/ODs/BMP-2 group had the highest ratio of dentin volume to total dentin volume, while the control group had the lowest ratio (Table 3).

### 3.9. Cell Tracking

**Figure 6:**
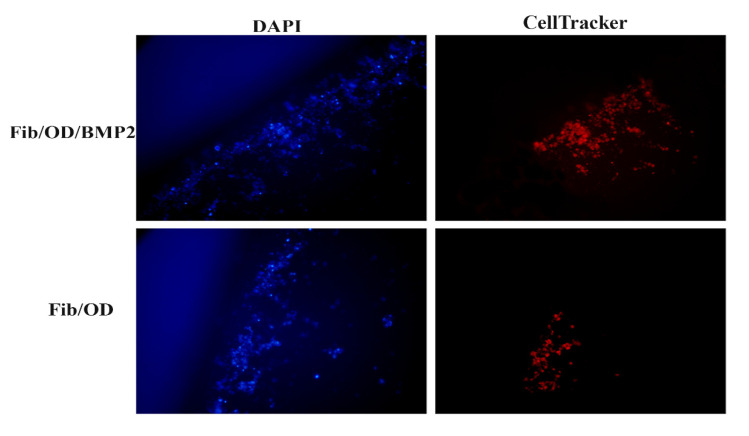
Cell tracking. C-EnSCs were labeled with DiI dye to follow homing of the transplanted cells in the injured site 8 weeks after transplantation. The slides were investigated under a fluorescent microscope, and the labeled cells were observable in red at the injured locus (10×).

C-EnSCs were labeled with DiI dye before transplantation to the injured pulp. Eight weeks after transplantation, the rats were euthanized, and the transplant site was checked for the presence of labeled cells using a fluorescent microscope. As shown in Figure 6, the marked cells were still detectable in red at the transplantation site, indicating the location of the cells at the zone and their participation in restoration and regeneration (Figure 6).

## 4. Discussion

Caries and minor fractures of teeth cause only slight inflammation of the pulp and surrounding tissues of the root at first and rarely are accompanied by pain. However, over time, irreversible damage to the pulp occurs (usually accompanied by pain), which leads to subsequent pulp necrosis and periapical diseases [2]. The primary goal in the treatment of permanent teeth with open apex is to preserve the life of the pulp through vital pulp therapy (VPT) for the occurrence of apicogenesis. Direct pulp capping and pulpotomy represent the VPT techniques used on open apex teeth for the natural development of tooth roots [20]. In recent years, the use of stem cells represented a new and possible technique for apexification and regenerative endodontics [21]. In dentistry, tissue engineering has proven to be a promising treatment option as researchers examine methods for restoring function and structure to diseased dental tissues [22]. However, their clinical relevance and applications depend on the regenerative outcomes. A critical aspect of achieving the desired outcomes regards stem cell selection and microenvironment design (allowing for favorable cell–cell, cell–biomaterial, and cell–signaling molecule interactions) [4]. Optimizing scaffold design and identifying the appropriate growth factors are crucial to optimally promote the odontogenic differentiation of SCs for dental tissue regeneration. However, the ideal scaffold structure and critical growth factor necessary for creating optimal microenvironments for dentin regeneration have not yet been determined [4,23].

### 4.1. The Endometrium Contains a Vascular Stroma with High Regeneration Capability

The endometrium is considered an attractive source of mesenchymal stem cells (MSCs) for cell-based therapy due to the easy accessibility of obtaining MSCs and their immunoregulatory properties [24,25]. EnSCs were observed to have a role in regenerative therapies, especially in immunodeficient mice with Duchenne muscular dystrophy [26]. Even though the exact mechanism remains not fully clarified, cell fusion and in situ differentiation could probably explain the efficacy of that kind of therapy. Indeed, the migration of EnSCs to muscle fibers was observed to enhance angiogenesis [27]. Moreover, EnSCs cells are used to treat Parkinson’s disease, bone regeneration, myocardial infarction, stroke, pelvic organ prolapses, tissue engineering applications, and skin wound healing in murine models [27,28,29]. EnSCs have also been used to restore the bladder wall without immunological side effects [30].

In this study, the C-EnSCs were isolated by the enzymatic method and identified by flow cytometry. Odontogenic differentiation into adipose, bone, and neural cells was assessed by alizarin red staining. The high expression of DMP1 and DSPP genes compared to the control group confirmed the significant differentiation of cells.

This study evaluated the dental regeneration by transplantation of odontoblasts derived from endometrial stem cells encapsulated in fibrin gel associated with BMP-2 in a rat direct pulp-capping model. Our results indicated that fibrin could increase cell viability, extension, and transition.

The MTT test was performed to determine the effects of BMP-2 on the viability of C-EnSCs, and a concentration of 100 ng/mL of BMP-2 was used for implantation. Then, histomorphologic and histomorphometric studies were conducted in different groups after 8 weeks. The obtained results showed dentin regeneration was highest in the fibrin/ODs/BMP-2 group compared to other groups and lowest in the control group. The stromal cells isolated from the uterine endometrium were able to adhere to plastic surfaces and differentiate into osteogenic, chondrogenic, and adipogenic tissue. Additionally, they expressed a panel of surface antigens commonly found in MSCs. Consequently, the isolated endometrial cells met the three criteria outlined by the “Mesenchymal and Tissue Stem Cell Committee of the International Society for Cellular Therapy”, which are the criteria for defining a selected cell population as multipotent mesenchymal stromal cells, including [18]:

MSC must be plastic-adherent when maintained in standard culture conditions;

MSC must express CD105, CD73 and CD90, and lack expression of CD45, CD34, CD14, or CD11b, CD79a or CD19, and HLA-DR surface molecules;

MSC must differentiate into osteoblasts, adipocytes and chondroblasts in vitro.

### 4.2. Differentiation of C-EnSCs

Our study found that C-EnSCs could also differentiate into neural, adipose, and adipocytes. This demonstrates the stem and potency of C-EnSCs, which can be differentiated into different types of mesenchymal lines. This study showed that these cells expressed surface markers CD90, CD73, and CD105 in 99.5%, 94.3%, and 99%, respectively.

On the other hand, in restoring the dentin–pulp complex, stem/progenitor cells were found in active tissues and migrated to the damaged area. These cells then differentiated into OD cells and subsequently formed dense tissue beneath the exposed pulp tissue [18]. The treated dentin matrix secreted a combination of soluble agents that induced stem cell differentiation into odontoblasts; MSCs were first differentiated into OD cells to promote mineralization and dentin formation [18,31]. Accordingly, we utilized OD cells derived from C-EnSCs to accelerate the process of dentin regeneration and ultimately preserve the life of teeth.

In studies to assess the odontogenic differentiation of stem cells, the expression of DMP-1 and DSPP was assessed. It was found that these genes were highly expressed in odontogenic differentiation [32,33,34]. The expression levels of DMP1 and DSPP genes were also examined in our study after odontogenic differentiation of C-EnSCs, both of which were significantly higher than the control group.

### 4.3. Role of Scaffolds in Tissue Repairments

Three-dimensional scaffolds and hydrogels alone or combined with bioactive molecules or cells can guide the development of functionally engineered tissues and supply mechanical support during in vivo implantation [35,36].

Although ceramic, metal, and polymeric scaffolds are commonly used in tissue engineering and grafting, hydrogel scaffolds are typically preferred to minimize cellular shocks caused by transfer and ensure proper cell survival. It has been found that a cell suspension based on higher viscosity biomass has a higher survival rate than a suspension based on saline [35]. In addition, when cells acquire the appropriate spatial structure in this viscosity and a three-dimensional scaffold, they are less likely to undergo apoptosis [37]. For this purpose, various hydrogels that are structurally identical to the extracellular matrix have been used, including collagen hydrogels, matrigels, self-aggregating peptides, chitosan, and alginate [9,35]. Therefore, fibrin-based scaffolds are considered one of the most suitable options because fibrin’s biological properties can be used to deliver specific drugs, growth factors, and cell lines [37]. As an example, the presence of peptide sequences with arginine–glycine–aspartic acid (RGD) within fibrin fibres induces cell–cell connection via integrin receptors [8]. Fibrin gel is also acknowledged for its high elasticity, making it a more effective cellular matrix than other proteins. Different types of cells have been injected using fibrin scaffolds to regenerate tissues of different organs, including the skin, bladder, trachea, cartilage, bone, and liver [9]. Additionally, fibrin can be used with natural and synthetic biological materials [10,36]. Our histological analysis found that the dentin bridge was incompletely formed in the fibrin group. Higher magnification revealed more dentin tubules, but no lacunae were detected. The odontoblast layer and odontoblast cells were not observed in this group. Furthermore, this group did not have calcification, but blood vessels appeared atrophic. On the other hand, loose connective tissue was formed in the pulp.

In the group that OD cells encapsulated in fibrin gel, dentin bridges had a reasonable consistency and thickness. On the other hand, odontoblast cells were found scattered in this group. Upon further investigation, dentin formation was observed along with tubules and lacunae. Calcification and the formation of blood vessels also occurred.

### 4.4. Growth Factors Role in Differentiation and Tissue Regeneration

The bone morphogenetic protein 2 has been demonstrated to be odontogenic and osteogenic both in vitro and in vivo [37]. BMP-2 has several functions: it promotes the differentiation of bovine, porcine, canine, and human dental pulp stem cells (DPSCs) into odontoblasts (resulting in dentin formation), induces dental follicle progenitor cells (DFPCs) to differentiate towards a cementoblast/osteoblast phenotype [4], and it can also stimulate odontogenesis of stem cells from human exfoliated deciduous teeth (SHED) and human tooth germ stem cells [38,39]. Nevertheless, the synergic effect of BMP-2 and OD cells derived from C-EnSCs on dental pulp regeneration has not been investigated until now. However, some studies have indicated that BMP-2 exhibits odontogenic and mineralizing properties [13,14].

A study evaluating the viability/differentiation of hDPSCs associated with BMP-2, TGF-β1, and BMP-2/TGF-β1 odontogenic differentiation was carried out for 14 days [14]. There was no difference in viability between the control group and the other groups (*p* > 0.05). BMP-2 was the major factor in the odontogenic differentiation of hDPSCs, which was further enhanced by co-stimulation with TGF-β1. Continuous stimulation with TGFβ-1 did not improve the differentiation of hDPSCs. They showed that alkaline phosphatase (ALP) activity and calcium content increased more in BMP-2-treated groups than in control groups. The expression levels of collagen type I (Col I), Bone sialoprotein (BSP), Osteocalcin (OCN), and DSPP genes in BMP-2 groups were significantly higher than in control groups. Furthermore, BMP-2 increased the expression of DMP-1 and Osteopontin (OPN) [4,14]. According to our study, BMP-2 at a concentration of 100 ng/mL had no toxic effects on C-EnSCs. The presented data show that OD cells treated with BMP-2 encapsulated in fibrin gel formed odontoblast cells in layers. Additionally, calcification occurred, and the blood vessels were fully developed. Dentin formation was observed along with tubular layers and lacunae. Moreover, no loose connective tissue or a very minimal amount was observed.

In a study, BMP-2 and the vascular endothelial growth factor (VEGF)-loaded three-dimensional model (TDM) for the enhanced angiogenic and odontogenic potential of dental pulp stem cells were investigated. It was found that TDM consisting of hydrogel and dentine matrix allowed cell–cell interactions. TDM was highly effective in delivering both BMP-2 and VEGF, which enhanced the angiogenic and odontogenic potential of DPSCs [40]. Synergistic effects of stromal cell-derived factor-1α (SDF-1α) and BMP-2 treatment on odontogenic differentiation of human stem cells from apical papilla (SCAP) cultured in the VitroGel 3D system were evaluated. The results showed that SCAP cultured in 3D hydrogel demonstrated favorable viability and proliferation. SDF-1α and BMP-2 cotreatment enhanced odontogenic differentiation-related gene and protein expression in vitro and promoted odontogenic differentiation of SCAP in vivo [41]. In our study, the fibrin/ODs/BMP-2 group showed the highest rate of angiogenesis and dentin regeneration performed by odontoblasts and BMP-2.

### 4.5. Strength and Limitations

The results of the present study show how the advanced techniques of tissue engineering, combining the use of mesenchymal cells and BMPs, can induce the proliferation and regeneration of dentine tissue after injuries, with potential application for the treatment of dental hard tissues.

Limits of the study include the use of an animal model and the difficulties of using these techniques in human clinical trials.

## 5. Conclusions

The results of our study have shown that direct pulp capping by OD cells derived from C-EnSCs has a promising capacity for dentin regeneration. Fibrin gel as a scaffold or carrier for the transfer of cells and BMP-2 as a growth factor have synergistic effects on each other, greatly enhancing the rate and quality of dentin regeneration. Therefore, OD cells derived from C-EnSCs encapsulated in fibrin gel associated with BMP-2 could be a promising approach for direct pulp capping and dentin regeneration.

## Figures and Tables

**Table 1:** Primers used for qRT-PCR.

Name	Primer Sequence (5′→3′)	Accession
ACTB (R)	TCGTCCCAGTTGGTGACGAT	NM_001101.5
ACTB (F)	GCATGGGTCAGAAGGATTCCT	
DSPP (R)	TTGCTTTGAGGAACTGGAAT	NM_014208.3
DSPP (F)	ATGCAAAAGTCCAGGACAG	
DMP1(R)	TTGATACCTGGTTACTGGGA	NM_004407.4
DMP1(F)	TTCTTTGTGAACTACGGAGG	

**Table 2:** Dentin bridge thickness (μm).

Case	Control	Fibrin	Fibrin/ODs	Fibrin/ODs/BMP-2
1	0.25	0.38	0.99	0.97
2	0.20	0.42	0.95	1.19
3	0.33	0.39	0.96	1.11
4	0.19	0.33	0.97	1.25
5	0.22	0.43	0.99	1.29
Mean ± DS	0.24 ± 0.14	0.39 ± 0.10	0.97 ± 0.04	1.16 ± 0.32

**Table 3:** The ratio of the volume of formed dentin to the total volume.

Case	Control	Fibrin	Fibrin/ODs	Fibrin/ODs/BMP-2
1	35	87	250	297
2	41	94	255	295
3	43	83	266	290
4	30	83	262	294
5	53	90	258	289
Mean ± DS	40 ± 13	87 ± 7	258 ± 16	293 ± 8

## Data Availability

The datasets generated during and/or analyzed during the current study are available from the corresponding author upon reasonable request.
